# Activation of Classical Brown Adipocytes in the Adult Human Perirenal Depot Is Highly Correlated with PRDM16–EHMT1 Complex Expression

**DOI:** 10.1371/journal.pone.0122584

**Published:** 2015-03-26

**Authors:** Gaku Nagano, Haruya Ohno, Kenji Oki, Kazuhiro Kobuke, Tsuguka Shiwa, Masayasu Yoneda, Nobuoki Kohno

**Affiliations:** Department of Molecular and Internal Medicine, Graduate School of Biomedical & Health Sciences, Hiroshima University, Hiroshima, Japan; University of Minnesota, UNITED STATES

## Abstract

Brown fat generates heat to protect against cold and obesity. Adrenergic stimulation activates the thermogenic program of brown adipocytes. Although the bioactivity of brown adipose tissue in adult humans had been assumed to very low, several studies using positron emission tomography–computed tomography (PET–CT) have detected bioactive brown adipose tissue in adult humans under cold exposure. In this study, we collected adipose tissues obtained from the perirenal regions of adult patients with pheochromocytoma (PHEO) or non-functioning adrenal tumors (NF). We demonstrated that perirenal brown adipocytes were activated in adult patients with PHEO. These cells had the molecular characteristics of classical brown fat rather than those of beige/brite fat. Expression of brown adipose tissue markers such as uncoupling protein 1 (UCP1) and cell death-inducing DFFA-like effector A (CIDEA) was highly correlated with the amounts of PRD1-BF-1-RIZ1 homologous domain-containing protein-16 (PRDM16) – euchromatic histone-lysine N-methyltransferase 1 (EHMT1) complex, the key transcriptional switch for brown fat development. These results provide novel insights into the reconstruction of human brown adipocytes and their therapeutic application against obesity and its complications such as type 2 diabetes.

## Introduction

Obesity, a serious global health problem, results from a chronic imbalance between energy intake and expenditure. Mammals have two distinct types of adipocytes: white adipocytes accumulate excessive energy as triglycerides, whereas brown adipocytes function to dissipate chemical energy as non-shivering thermogenesis via uncoupling protein 1 (UCP1) expression [1]. Brown adipocytes are abundant in rodents and human infants to defend against cold. In adult humans, brown adipocytes were thought to be nonexistent or present without physiologic function. In 2009, several studies using ^18^F-fluorodeoxyglucose (FDG) positron emission tomography–computed tomography (PET–CT) reported that normal adult humans under cold exposure possess bioactive brown adipose tissue (BAT) [2–5]. Moreover, the amount of BAT in adult humans is inversely correlated with obesity and age [2, 5], and BAT dysfunction leads to obesity and insulin resistance in experimental models [6, 7]. This evidence suggests that BAT plays an important role in energy metabolism; therefore, the activation of BAT has attracted much attention as a novel therapeutic target for the treatment of obesity and its various complications.

According to past studies in rodents, brown adipocytes can be classified into at least two distinct types. “Classical” brown adipocytes exist primarily in rodent interscapular and perirenal regions, and show characteristic expression of genes such as early B cell factor-3 (*Ebf3*), F-box protein 31 (*Fbxo31*) and LIM Homeobox 8 (*Lhx8*) [8–10]. So-called “beige” or “brite” adipocytes are found dispersed in white adipose tissue (WAT). Beige adipocytes express characteristic genes such as cluster of differentiation-137 (*Cd137*) (also known as tumor necrosis factor receptor superfamily member 9 (*Tnfrsf9*)), T-box 1 (*Tbx1*), and transmembrane protein 26 (*Tmem26*) [8, 10]. They are induced under cold exposure or adrenergic stimulation by catecholamines in experimental models [10–12]. Some reports have shown that beige-selective markers are present in human BAT [10, 11], and others have reported that human BAT in the interscapular and deep neck regions exhibits the same gene profiles as murine classical brown adipocytes [8, 13]. A few studies have demonstrated that human BAT in the perirenal region has the same activity as that in the interscapular or supraclavicular areas [14, 15]. Very few studies have investigated the lineage of human perirenal BAT owing to the difficulties associated with collecting human samples. The lineage of human perirenal BAT remains unclear.

Pheochromocytoma (PHEO) is defined as a neuroendocrine tumor that produces and secretes large amounts of catecholamines such as epinephrine (adrenalin) or norepinephrine (noradrenalin). Excessive levels of catecholamines cause a variety of hemodynamic and metabolic symptoms including body weight loss independent of food intake or exercise [16]. In patients with PHEO, resting energy expenditure, an index of energy metabolism, is decreased by adrenalectomy [17]. Some studies using PET–CT in PHEO patients have shown intense FDG uptake in the supraclavicular and perirenal regions without cold exposure, and this effect disappears after tumor excision [18, 19]. This phenomenon may reflect BAT activation via excessive catecholamine levels in patients with PHEO.

A subset of myoblast-like precursor cells positive for myogenic factor 5 (*Myf5*) differentiates into brown adipocytes during embryological development [20]. PR domain-containing protein-16 (PRDM16) is a key transcriptional regulator that induces BAT in rodents [7, 9, 21]. The expression of *PRDM16* has been detected in supraclavicular adipose tissue in adult humans, corresponding to similar tissues in rodents [13]. We previously identified euchromatic histone-lysine *N*-methyltransferase 1 (EHMT1) as an essential brown fat-enriched methyltransferase that controls the specification and maintenance of brown adipose cell fate along with PRDM16 [6]. Interestingly, mutations in the human EHMT1 gene are often associated with obesity [22]. However, there is a lack of research investigating whether the PRDM16–EHMT1 complex serves as a key factor in the development of human brown adipocytes.

In this study, we demonstrated the existence and properties of classical brown adipocytes in the perirenal region of adult patients with PHEO. Furthermore, the amount of the PRDM16–EHMT1 complex was positively correlated with the expression levels of BAT-associated genes.

## Materials and Methods

### Subjects and samples

Eleven subjects with adrenal PHEO and seven subjects with non-functioning adrenal tumor (NF) were enrolled in this study. Subject characteristics were matched by age and gender (Table 1). Endocrinological diagnoses were performed following our previously reported methods [23]. All of the subjects underwent laparoscopic adrenalectomy and chunks of adipose tissues were simultaneously dissected from the perirenal fat depots and snap-frozen at -80°C. The diagnosis of PHEO was confirmed by pathological findings. The levels of preoperative urinary catecholamines (epinephrine and norepinephrine) were analyzed in pooled urine for 24 hours (SRL, Tokyo, Japan).

**Table 1:** Subject characteristics.

Characteristic	NF	PHEO	***P***-value
**Number (male/female)**	7 (2/5)	11 (4/7)	—
**Age (years)**	50.7 ± 5.0	52.2 ± 4.8	0.834
**BMI (kg/m** ^**2**^ **)**	28.5 ± 2.1	21.0 ± 1.0	0.011
Total preoperative urinary catecholamines* (μg/day)	103.8 (87.2–174.0)	543.0 (303.8–1720.5)	0.001

Comparisons between the NF and PHEO groups.

Values are the means ± SEM, median (interquartile range), number, or *P*-value.

*Values were normalized by logarithmic conversion before comparisons.

### RNA isolation and gene expression analysis

Total RNA was isolated from tissues using TRI Reagent (Molecular Research Center, Cincinnati, OH, USA) or TRIzol Reagent (Invitrogen, Carlsbad, CA, USA), according to the manufacturers’ protocols. Reverse transcription was performed using PrimeScript RT Master Mix (Perfect Real Time) (TaKaRa Bio, Ohtsu, Japan). Quantitative real-time PCR (qRT-PCR) was performed with SYBR Green fluorescent dye using the 7500 Fast PCR Machine (Applied Biosystems, Foster City, CA, USA). TATA-binding protein (TBP) served as an internal control. Primer sequences are shown in S1 Table. The expression levels were calculated by the ΔΔC_T_ method, and all derived data were normalized by logarithmic transformation (base = 10) for each analysis.

### Western blot analysis

Samples were lysed in lysis buffer (50 mM Tris-HCl, pH 7.4, 150 mM NaCl, 10% glycerol, 100 mM NaF, 10 mM EGTA, 1 mM Na_3_VO_4_, 1% Triton X-100, 5 μM ZnCl_2_ with a 1/100 dilution of protease inhibitor cocktail (Sigma Aldrich, St. Louis, MO, USA)). The amount of protein was quantified using the Pierce BCA Protein Assay Reagent Kit (Thermo Scientific, Waltham, MA, USA) according to the manufacturer’s protocol, and 20 μg of total protein was applied and separated by sodium dodecyl sulphate-polyacrylamide gel electrophoresis (SDS-PAGE) and transferred to a polyvinylidene difluoride (PVDF) membrane (Millipore, Billerica, MA, USA). Antibodies for UCP1 (Abcam, # ab10983, Cambridge, UK), cell death-inducing DFFA-like effector A (CIDEA, Sigma Aldrich, # PRS2085), EBF3 (Millipore, # AB10525), PRDM16 (Abcam, # ab118573), EHMT1 (Perseus Proteomics, # AB205007, Tokyo, Japan) and βActin (Cell Signaling Technology, # 4967, Danvers, MA, USA) were used for probes. Quantification of the bands was assessed with ImageJ version 1.47 (National Institutes of Health, Bethesda, MD, USA). Values obtained were normalized logarithmically (base = 10) for each analysis.

### Histology and immunohistochemistry

Samples were cut in 10 μm sections at -20°C using a cryomicrotome (Leica, Wetzlar, Germany) and mounted on silane-coated glass slides (Muto Pure Chemicals, Tokyo, Japan). Sections were fixed in ice-cold acetone. Hematoxylin and eosin (H&E) staining was performed according to standard procedures. For immunohistochemistry, frozen sections were incubated with anti-UCP1 (Abcam, # ab10983), anti-CIDEA (Sigma-Aldrich, # PRS2085), or anti-EBF3 (Millipore, # AB10525). Secondary detection was performed using anti-rabbit Histofine Simple Stain MAX PO (Nichirei Bioscience, Tokyo, Japan). These series of operations were performed by Kyodo Byori (Kobe, Japan). All observations were performed with a KEYENCE BZ-9000 (Keyence, Tokyo, Japan).

### Statistics and heat map

Comparisons between subject groups were assessed by unpaired two-tailed *t*-tests throughout the study. Correlations were assessed using Pearson’s correlations. The reliability of correlations was analyzed by Cronbach’s α. *P* < 0.05 was defined as statistical significance. All statistical analyses including heat map generation were performed using R version 2.15.3 [24].

### Study approval

The protocol for the study was approved by the Hiroshima University Ethics Committee (Hiroshima, Japan). All subjects gave written informed consent before participating in the study. All procedures were conducted according to the principles of the Declaration of Helsinki.

## Results

### Perirenal BAT is more apparent in adult patients with PHEO

Subject characteristics are shown in Table 1 and S2 Table. All subjects were over 30 years of age (ranging from 30 to 86 years) and the mean age was 52.2 ± 4.8 in subjects with PHEO and 50.7 ± 5.0 in those with NF (*P* = 0.83). Subjects with PHEO were leaner than those with NF (*P* = 0.011), and total preoperative urinary catecholamine (the sum of epinephrine and norepinephrine) levels were higher in PHEO subjects than in NF subjects (*P* = 0.001).

**Fig 1:**
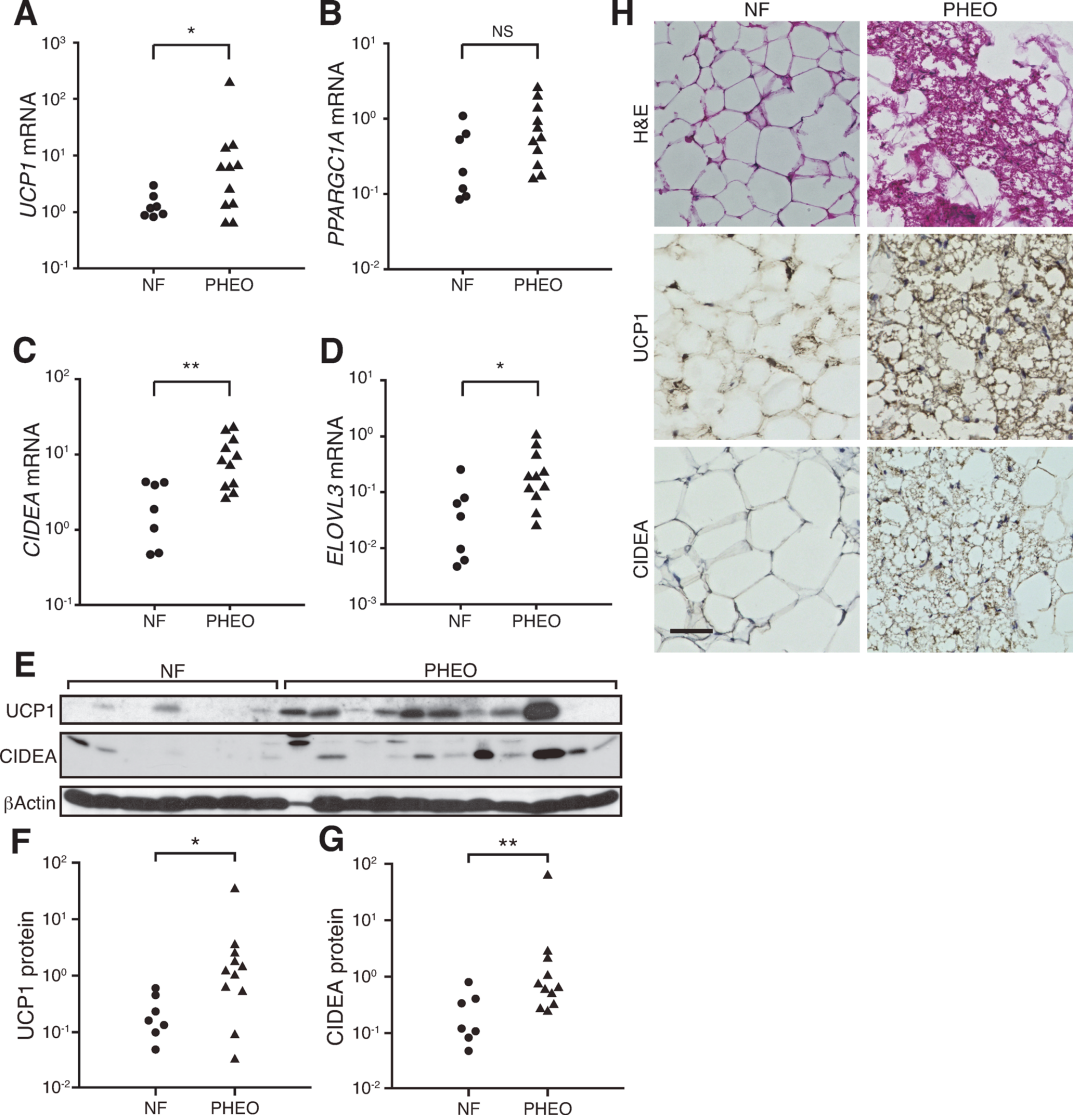
Brown adipose tissues are activated in the perirenal regions of PHEO patients. Comparisons of (A–G) BAT-associated mRNA (A, *UCP1*; B, *PPARGC1A*; C, *CIDEA*; D, *ELOVL3*) and protein (F, UCP1; G, CIDEA) between NF and PHEO in adipose tissues from the perirenal regions. (E) Protein quantification was performed using western blot analysis. (H) H&E staining (top) and immunohistochemistry using antibodies against UCP1 (middle) and CIDEA (bottom). Scale bar, 50 μm. *P < 0.05; **P < 0.01; NS, not significant.

We assessed mRNA levels of well-known BAT-associated gene markers such as *UCP1*, peroxisome proliferator-activated receptor gamma coactivator 1 alpha (*PPARGC1A*), *CIDEA*, and elongation of very long chain fatty acids-like 3 (*ELOVL3*) in adipose tissues from the perirenal regions. Not only the cAMP-inducible thermogenic gene (*UCP1*), but also the BAT-associated genes that are not sensitive to temporary cAMP elevation (*CIDEA* and *ELOVL3*) [25] showed significantly higher expression in PHEO subjects than in NF subjects (*P* = 0.029, 0.005, and 0.015, respectively) (Fig. 1A–D). These genes were previously reported to be linked to BAT activity [21]. Furthermore, the expression levels of both UCP1 and CIDEA were significantly higher in PHEO subjects at the protein level (*P* = 0.018 and 0.009, respectively), as assessed by western blot analysis (Fig. 1E–G).

To confirm the existence of BAT in PHEO subjects, we performed H&E staining and immunohistochemistry for UCP1 and CIDEA. In PHEO samples, both UCP1 and CIDEA-positive multilocular brown adipocytes were observed, whereas positive cells for either protein were rare in NF samples (Fig. 1H). These results suggest that the stimulation by elevated catecholamines can activate adult human BAT, at least in the perirenal regions.

### Activated perirenal BAT in adult humans possess the molecular signatures of classical brown adipocytes

**Fig 2:**
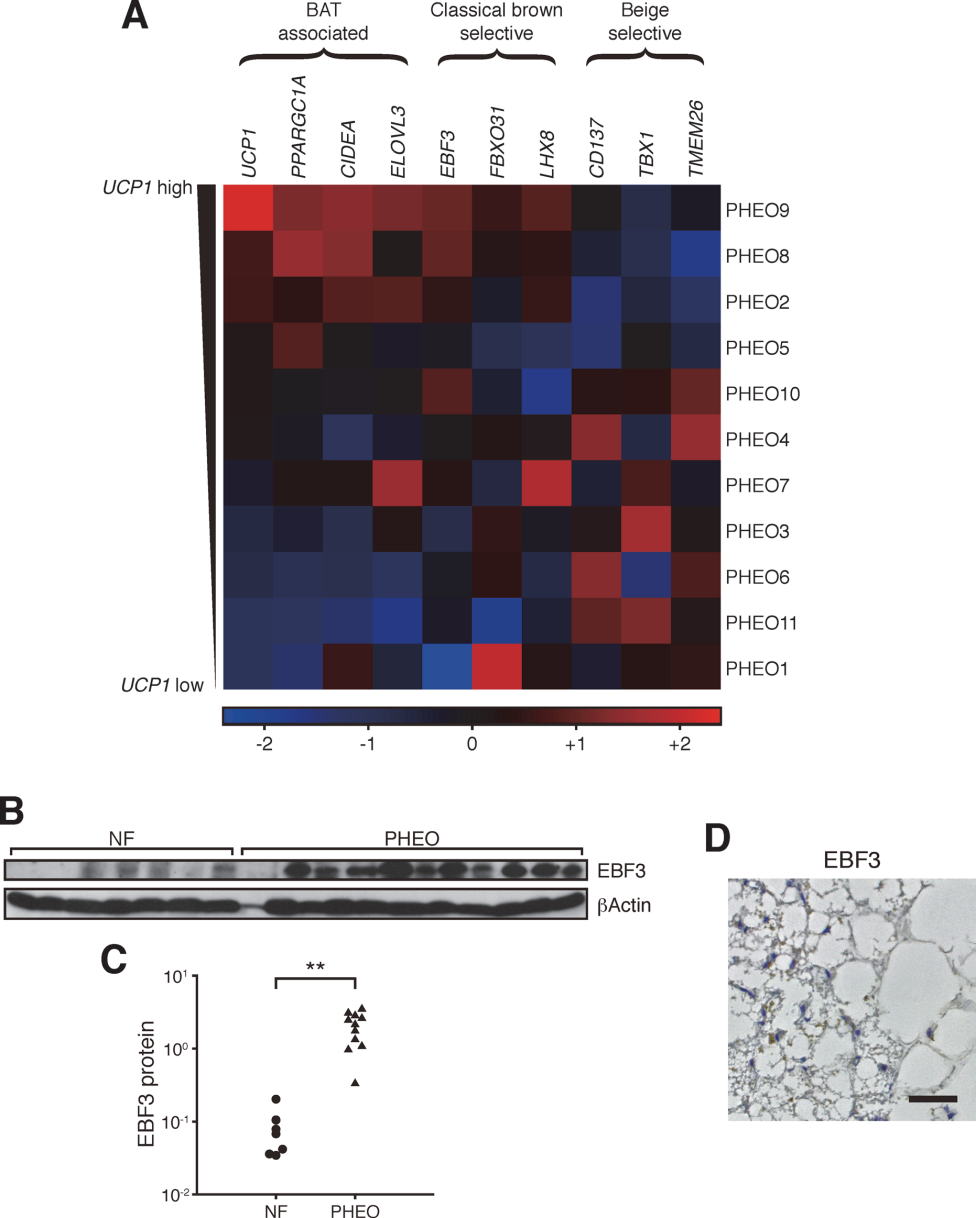
Activated BAT in the perirenal region has the molecular signatures of classical brown fat. (A) The heat map matrix of gene expression profiles in PHEO subjects. Subjects are arranged in order of *UCP1* mRNA expression levels. All data are normalized within each column using the Z-score method. The color scale shows the Z-score; the highest value is bright red, the lowest is bright blue, and the midpoint is black. (C) Comparison of EBF3 protein expression levels between NF and PHEO subjects in perirenal adipose tissues. The protein expression levels were assessed by western blot analysis (B). (D) Representative example of immunostaining for EBF3 in a PHEO sample. Scale bar, 50 μm. *P < 0.05; **P < 0.001, NS, not significant.

We investigated the lineage of the perirenal BAT in our samples. Classical brown selective markers showed significantly higher expression in PHEO samples than in NF samples, but the expression levels of beige-selective markers were not high in PHEO samples (S1 Fig.). Moreover, as shown in Fig. 2A, samples of PHEO patients with higher expression of BAT-associated markers showed higher expression of classical brown selective markers, such as *EBF3*, *FBXO31*, and *LHX8*, but lower expression of beige-selective markers including *CD137*, *TBX1*, and *TMEM26*. These results indicate that *EBF3*, *FBXO31* and *LHX8*, representative markers for classical BAT, may be more suitable markers for perirenal BAT in our samples than well-known beige adipocyte markers. Western blot analysis showed that expression of the EBF3 protein was significantly higher in PHEO subjects than in NF subjects (*P* < 0.001) (Fig. 2B and C). Furthermore, brown adipocytes showed distinct EBF3 staining (Fig. 2D). Hence, the perirenal BAT in adult humans has the molecular signatures of classical brown adipocytes rather than beige adipocytes, similar to murine perirenal BAT.

### The PRDM16–EHMT1 complex might play an important role in brown adipose cell development in adult humans

**Fig 3:**
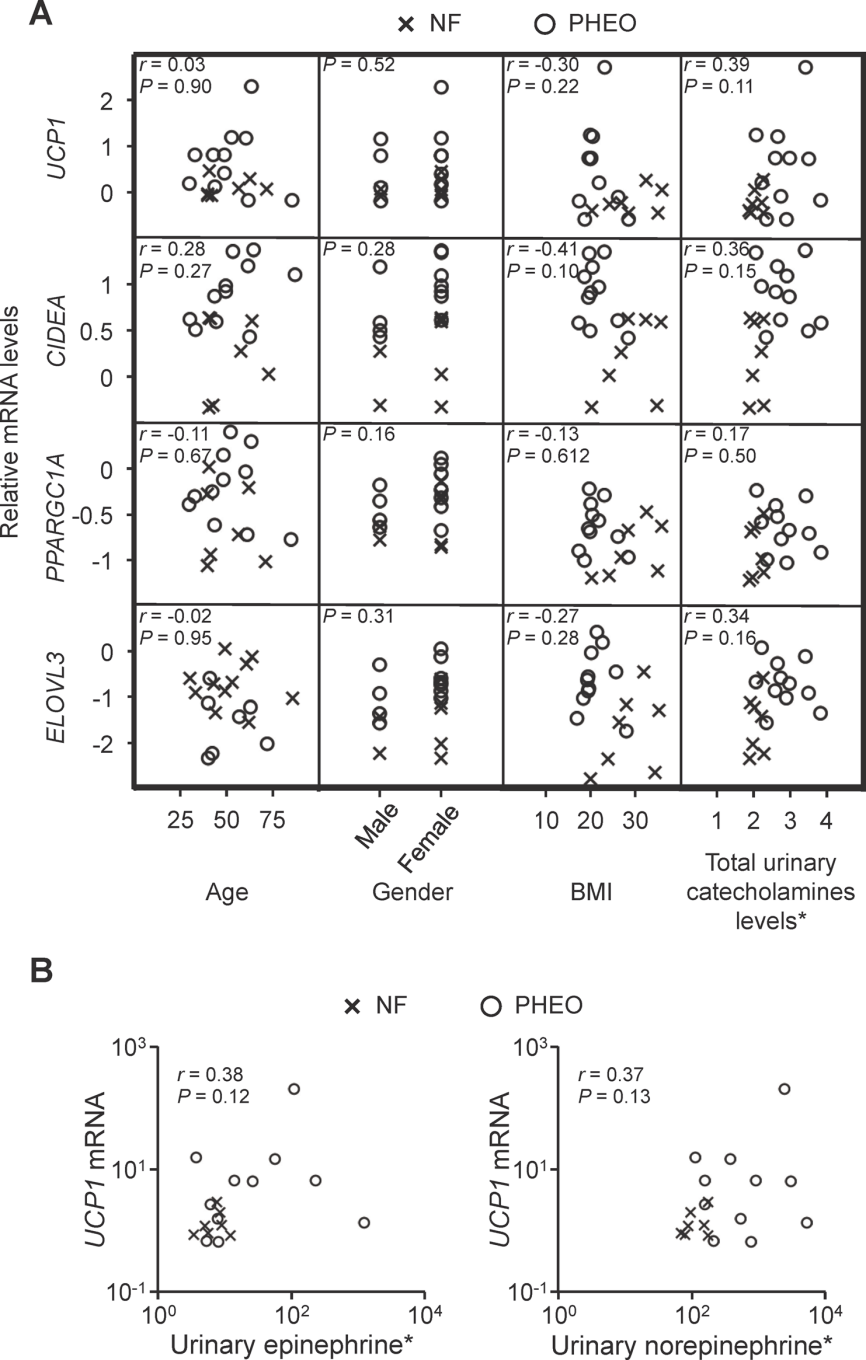
The expression levels of BAT-associated genes are not affected by clinical characteristics. (A) The correlation matrix between BAT-associated gene expression (top to bottom: UCP1, CIDEA, PPARGC1A, or ELOVL3) and subject characteristics (left to right: age, gender, body mass index (BMI), and total preoperative urinary catecholamine levels) in patients with NF (cross) or PHEO (circle). (B) Correlation analysis between individual urinary catecholamines and *UCP1* mRNA. *Values were normalized by logarithmic transformation before each correlation analysis.

Even in the PHEO group, some subjects expressed low levels of BAT-associated markers (Fig. 1). Therefore, we investigated whether any clinical characteristics affect the expression levels of BAT-associated genes. Although age and body mass index (BMI) are known to be inversely correlated with BAT activity [2, 5], there were no correlations between the BAT-associated genes and age or BMI (Fig. 3A). PHEO subjects tended to have higher levels of BAT-associated genes and urinary catecholamines compared to NF subjects. However, contrary to our expectation, no correlations were observed between BAT-associated genes and the total urinary catecholamine levels (Fig. 3A). Detailed analysis to assess the individual contribution of catecholamines showed no correlation between the expression levels of *UCP1* mRNA and urinary epinephrine or norepinephrine individually (Fig. 3B). In addition, there were no significant gender differences in the expression levels of BAT-associated markers (Fig. 3A).

**Fig 4:**
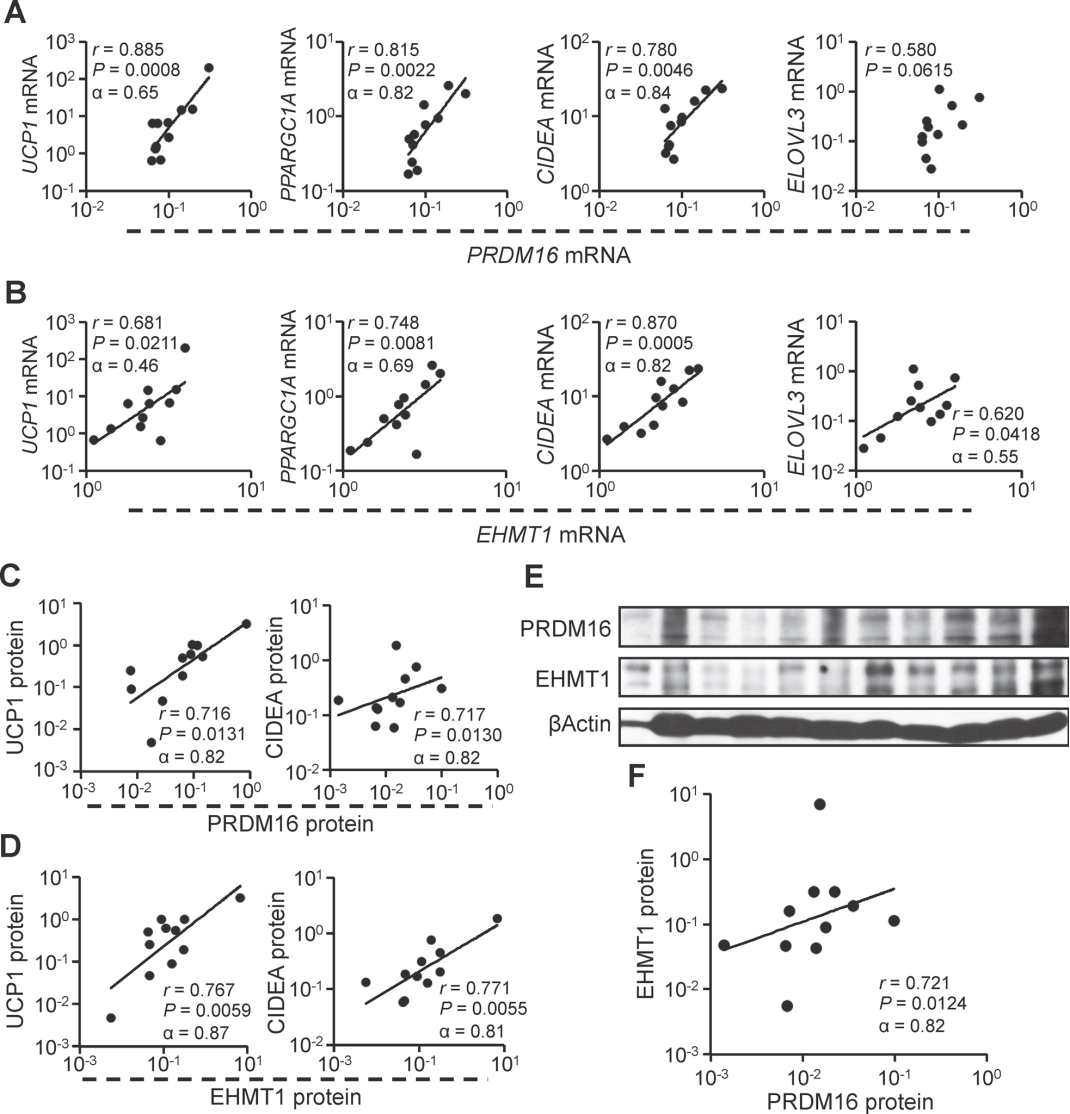
The PRDM16–EHMT1 complex plays an important role in the adult human brown adipose cell development. (A, B) Correlation analyses at the mRNA level were performed in tissues from patients with PHEO between *PRDM16* (A) or *EHMT1* (B) and BAT-associated genes (left to right, *UCP1*, *PPARGC1A*, *CIDEA*, and *ELOVL3*). (C, D) The correlation was also assessed at the protein level between PRDM16 and UCP1 (C, left), PRDM16 and CIDEA (C, right), EHMT1 and UCP1 (D, left), or EHMT1 and CIDEA (D, right). Protein expression levels were assessed by western blot analysis (shown in Fig. 1E and 3E). (F) Correlation between the PRDM16 protein and EHMT1 protein in PHEO samples. The protein expression levels were assessed by western blot analysis (E).

However, the expression of both PRDM16 and EHMT1 was positively correlated with that of BAT-associated markers (Fig. 4A and B). Although Cronbach’s α, the reliability of these correlations, was not very high owing to small numbers, they were statistically significant. The same correlations were observed at the protein level by western blot analysis (Fig. 4C and D). We analyzed the levels of these two proteins in NF and PHEO samples to investigate the effect of catecholamines. Even though the expression levels of PRDM16 or EHMT1 mRNA were high in PHEO samples (*P* = 0.062 and 0.014, respectively) (S2 Fig.), there was no significant difference between the two groups at the protein level (P = 0.100 and 0.465, respectively) (S2 Fig.). Furthermore, a positive correlation between PRDM16 and EHMT1 was observed (Fig. 4E and 4F), consistent with our previous report showing that EHMT1 stabilizes PRDM16 via direct interaction in mice [6]. These results suggest that the PRDM16–EHMT1 complex might play an important role in brown adipose cell development in adult humans.

## Discussion

β3 adrenergic agonists stimulate the thermogenic program of BAT via the adrenoreceptor-cAMP pathway [26]. From some decades ago, the existence of human BAT has been shown in patients with PHEO [27, 28, 29]. Recent studies have shown that the browning of WAT was observed in patients with PHEO [30, 31]. Thus many researchers have attempted to apply β3 adrenergic agonists to therapeutic regimens for obesity. However, they have not been effective remedies for obesity owing to pharmaceutical problems [32, 33]. Indeed, Cypess et al. have shown that an oral selective β3 adrenergic agonist can activate human BAT thermogenesis, but heart rate and blood pressure were significantly increased by the β3 adrenergic agonist [34]. In this study, subjects with PHEO were obviously leaner than those with NF. Moreover, genetic and morphological analyses indicate that stimulation with elevated catecholamines can activate the perirenal BAT in adult humans. Targeting the adrenoreceptor-cAMP pathway with avoiding undesirable heart side effects may be an appealing intervention to treat obesity.

Spiegelman and colleagues assessed the human BAT lineage and concluded that supraclavicular BAT consists of beige adipocytes [10]. Sharp et al. previously reported that human BAT from multiple locations, including subcutaneous supraclavicular areas, posterior mediastinum, retroperitoneal, intra-abdominal, mesenteric depots, and thigh tissues, exhibit signatures of beige adipocytes [11]. Jespersen et al. demonstrated that human BAT in the deep neck or supraclavicular regions has the molecular signatures of classical brown adipocytes [13]. Previous research investigating the lineage of human perirenal BAT has also concluded that brown adipocytes in the perirenal region possess the molecular signatures of beige adipocytes [35]. In this study, perirenal BAT in PHEO patients was surrounded by WAT. In this condition, it is easy to predict that excessive catecholamines might induce the browning of WAT. Although our study is limited to molecular analyses, our results support that the activated perirenal BAT in adult humans has the molecular signatures of classical brown adipocytes rather than beige adipocytes, similar to murine perirenal BAT. Another recent study by Svensson et al. supports that human perirenal BAT does not have the molecular marker patterns of typical beige adipocytes [36].

Furthermore, our results show the possibility that human classical brown adipocytes are activated by certain stimulants such as catecholamines, even in adults. The induction of beige cells and the activation of classical brown adipocytes should be considered as targets for human BAT studies. However, previous studies have pointed out the possibility that some murine markers discriminating the lineage of brown or beige adipocytes might be unsuitable for the classification of human BAT [13, 35, 37]. There might be brown adipocyte types in humans other than murine classical brown or beige adipocytes [35].

Ideally, the perirenal BAT should be obtained separately from WAT. The mixed brown and white fat could cause the correlations owing to tissue homogeneity. However, in reality, it is difficult to obtain pure BAT from surrounding WAT, especially in human samples. Direct histological confirmation of BAT by rapid pathological diagnoses might resolve this issue. Further assessment of the lineage of human perirenal BAT is required.

PRDM16 is the key transcriptional switch to induce brown fat from *Myf5*-positive precursors, which can also differentiate into skeletal muscle [9, 21]. Our previous study revealed that PRDM16 induces white-to-brown fat conversion in response to peroxisome proliferator-activated receptor gamma (PPARγ) agonists in mice [38]. In addition, we previously demonstrated that EHMT1 is an essential methyltransferase in the PRDM16 complex, and EHMT1 expression positively regulates the BAT-selective thermogenic program by stabilizing the PRDM16 protein [6]. We found a positive correlation between the expression levels of PRDM16 as well as EHMT1 and BAT-associated markers at both the mRNA and protein levels. Moreover, expression of the PRDM16 protein was positively correlated with EHMT1 protein expression. The current study is an observational investigation, but our results raise the possibility that these two proteins act in a coordinate manner in humans.

In conclusion, we demonstrated that perirenal brown adipocytes possessing the molecular signatures of classical BAT were activated in adult humans, and this activation was correlated with the amount of the PRDM16–EHMT1 complex. Future studies will investigate how to stabilize the PRDM16–EHMT1 complex and activate classical brown adipocytes in adult humans, which may lead to novel therapeutic strategies against obesity and metabolic diseases such as type 2 diabetes.

## Supporting Information

S1 Fig Classical brown selective markers are higher in samples of PHEO. The expression levels of classical brown selective markers including (A) *EBF3* and (B) *FBXO31* and beige-selective markers including (C) *CD137*, (D) *TBX1* and (E) *TMEM26* were analyzed by qRT-PCR in samples of both NF and PHEO. *P < 0.05; NS, not significant.
(TIFF)

S2 Fig The expression comparisons of PRDM16 and EHMT1 between NF and PHEO. PRDM16 and EHMT1 expression levels were analyzed at the mRNA (A) and protein level (B, lower panel; left, PRDM16; lower right, EHMT1) in the perirenal adipose tissues derived from patients with NF or PHEO. Protein levels were assessed using western blot analysis (B, upper panel). *P < 0.05; NS, not significant.
(TIFF)

S1 Table Primer sequences used for real-time PCR. (TIF)

S2 Table Detailed subject characteristics. (TIF)
