# Supplementary figures and images for: Activation of Classical Brown Adipocytes in the Adult Human Perirenal Depot Is Highly Correlated with PRDM16–EHMT1 Complex Expression
Source: PLoS One. 2015 Mar 26;10(3):e0122584. doi: 10.1371/journal.pone.0122584 (PMC4374757; doi:10.1371/journal.pone.0122584)

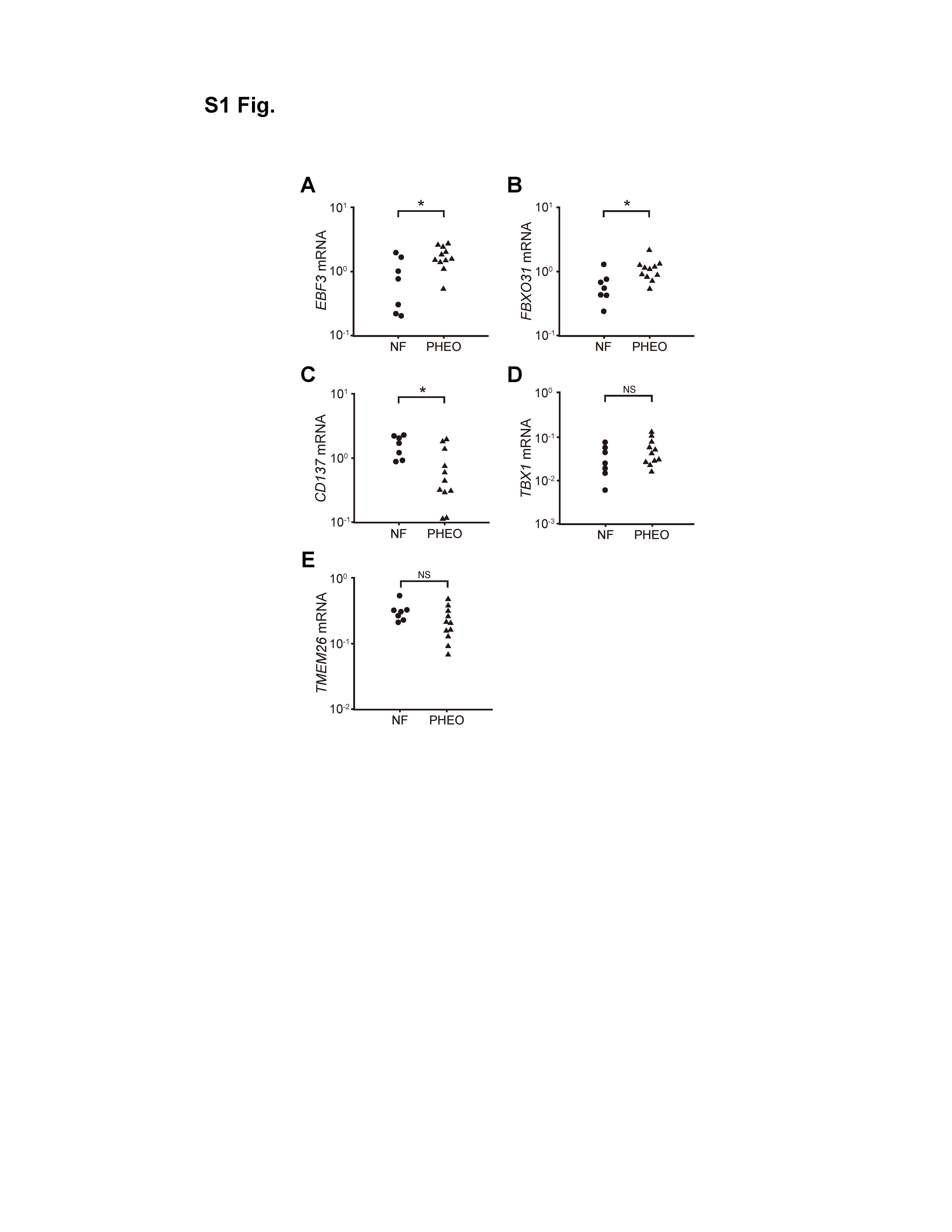

Supplement: S1 Fig — The expression levels of classical brown selective markers including (A) EBF3 and (B) FBXO31 and beige-selective markers including (C) CD137, (D) TBX1 and (E) TMEM26 were analyzed by qRT-PCR in samples of both NF and PHEO. *P < 0.05; NS, not significant. (TIFF) [file pone.0122584.s001.tiff]

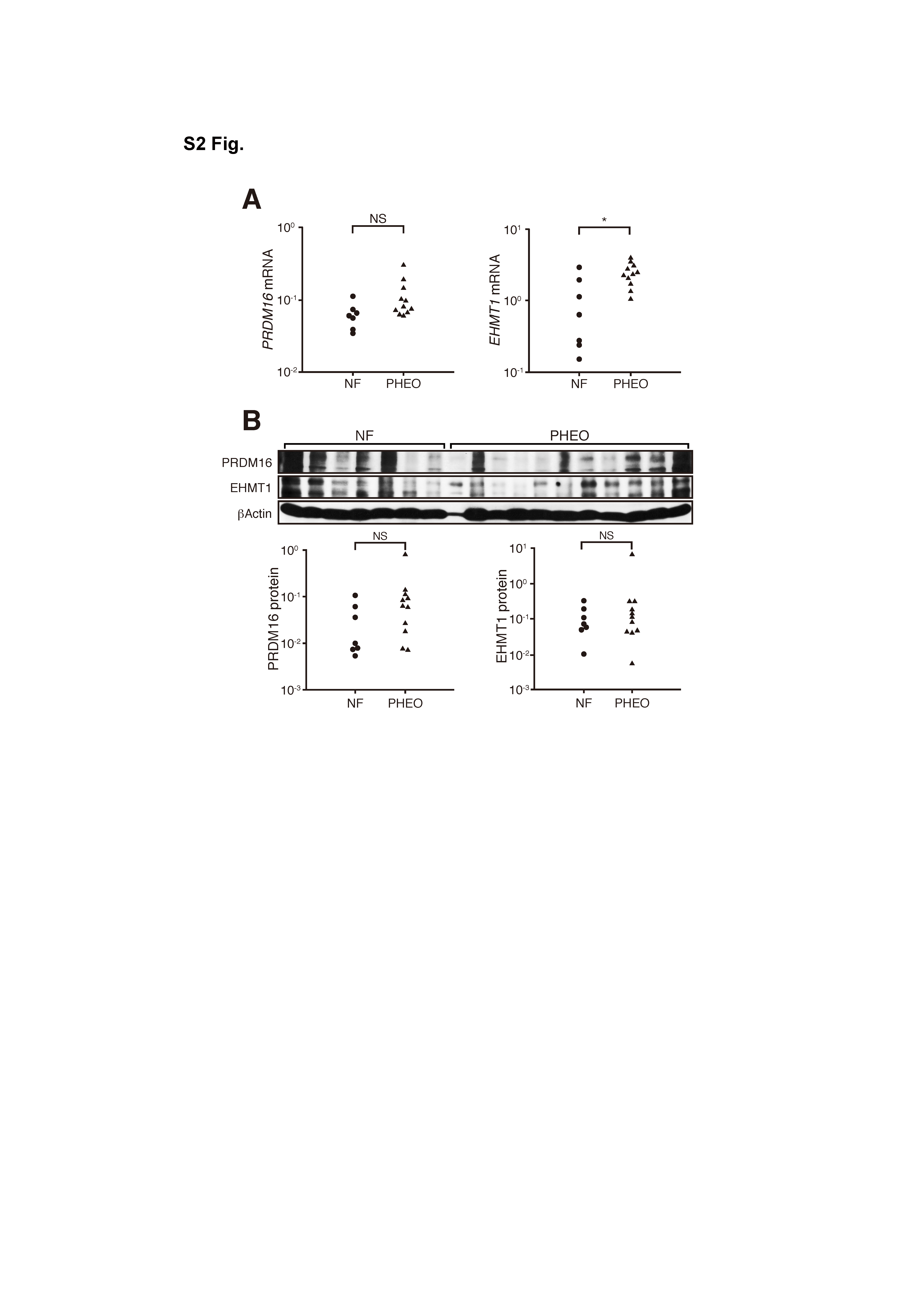

Supplement: S2 Fig — PRDM16 and EHMT1 expression levels were analyzed at the mRNA (A) and protein level (B, lower panel; left, PRDM16; lower right, EHMT1) in the perirenal adipose tissues derived from patients with NF or PHEO. Protein levels were assessed using western blot analysis (B, upper panel). *P < 0.05; NS, not significant. (TIFF) [file pone.0122584.s002.tiff]

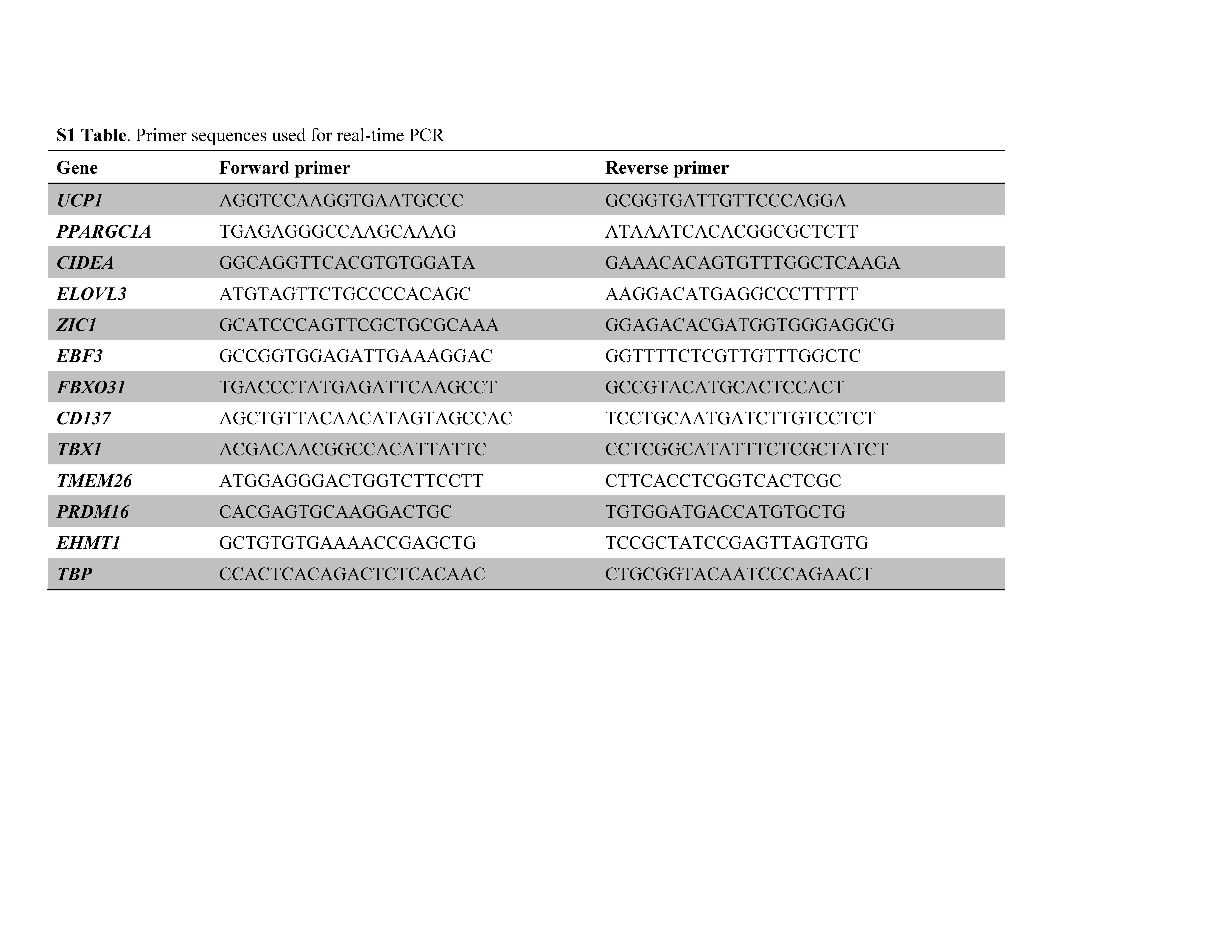

Supplement: S1 Table — (TIF) [file pone.0122584.s003.tif]

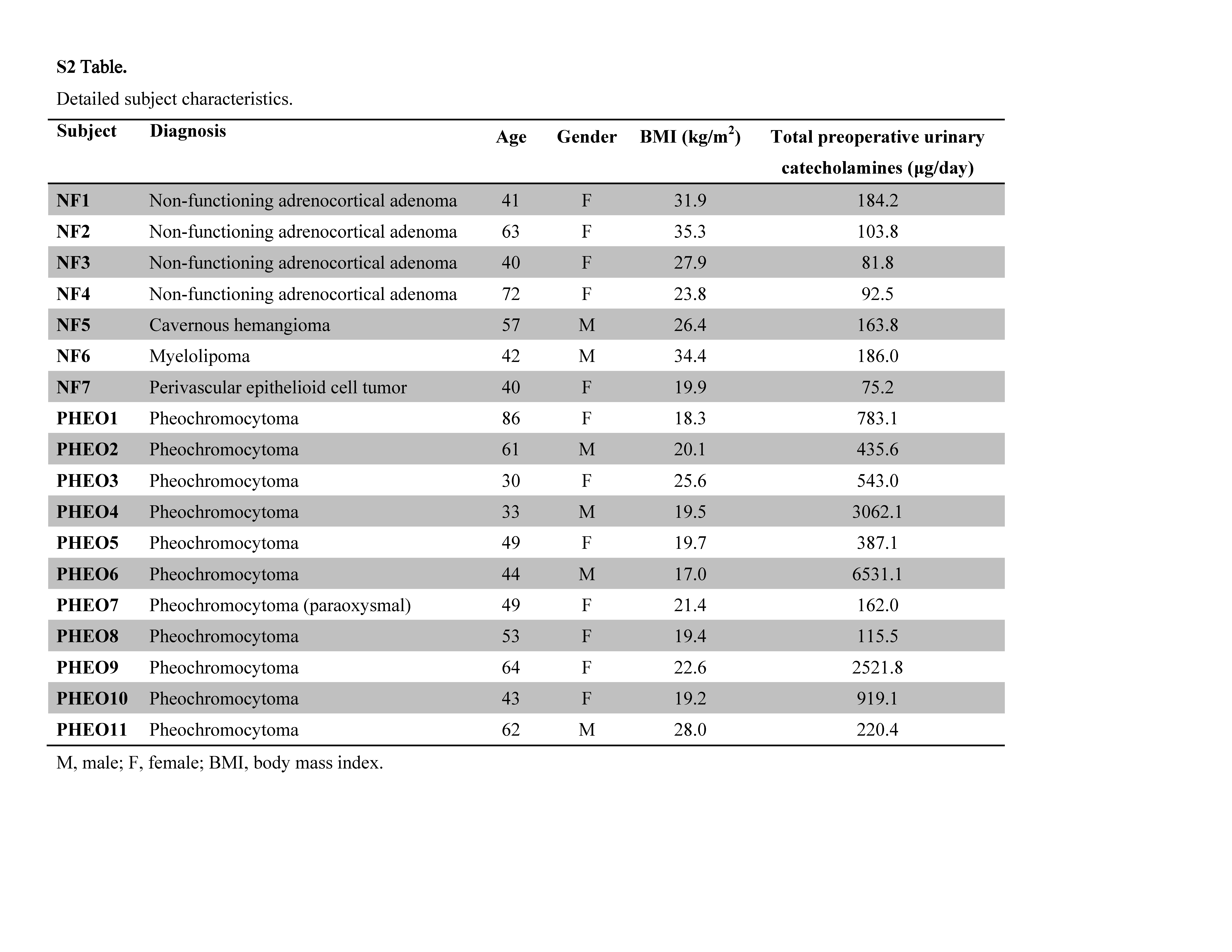

Supplement: S2 Table — (TIF) [file pone.0122584.s004.tif]
